# Sedative-hypnotic initiation and renewal at discharge in hospitalized older patients: an observational study

**DOI:** 10.1186/s12877-018-0972-3

**Published:** 2018-11-14

**Authors:** Elsa Bourcier, Amandine Baptiste, Adrien Borowik, Lucas Zerbib, Dominique Bonnet-Zamponi, Florence Tubach, Christine Fernandez, Patrick Hindlet

**Affiliations:** 1 Sorbonne Université, INSERM, Institut Pierre Louis d’épidémiologie et de Santé Publique, IPLESP UMR-S1136, F-75012 Paris, France; 2 Assistance Publique – Hôpitaux de Paris, Hôpital Saint-Antoine, Service de Pharmacie, 184, rue du Faubourg Saint Antoine, F-75012 Paris, France; 3 Université Paris-Sud, Faculté de Pharmacie, 5, rue Jean-Baptiste Clément, F-92296 Chatenay-Malabry, France; 4 AP-HP, Hôpital Pitié-Salpêtrière, Département Biostatistique Santé Publique et Information Médicale, Centre de pharmacoépidémiologie (Céphépi), INSERM, CIC-P 1421, 75013 Paris, France; 5 AP-HP, Hôpital Pitié-Salpêtrière, Centre de pharmacoépidémiologie (Céphépi), INSERM, UMR 1123, CIC-P 1421, 75013 Paris, France; 6 Observatoire du Médicament des Dispositifs Médicaux et de l’Innovation Thérapeutique Ile de France, Paris, France; 7 Sorbonne Université, Faculté de médecine Sorbonne Université, AP-HP, Hôpital Pitié-Salpêtrière, Département Biostatistique Santé Publique et Information Médicale, Centre de pharmacoépidémiologie (Céphépi), INSERM, UMR 1123, CIC-P 1421, 75013 Paris, France

**Keywords:** Sedative-hypnotics, Initiation, Older adults, Hospitalization, Risk factors

## Abstract

**Background:**

Sedative-hypnotics (SHs) are widely used in France but there are no available data addressing their prescription specifically in hospitalized older patients. The objective is thus to determine the cumulative incidence of sedative-hypnotic (SH) medications initialized during a hospital stay of older patients, the proportion of SH renewal at discharge among these patients and to study associated risk factors.

**Methods:**

We conducted a retrospective observational study in six internal medicine units and six acute geriatric units in eight hospitals (France). We included 1194 inpatients aged 65 and older without SH medications prior to hospitalization. Data were obtained from patients’ electronic pharmaceutical records. Primary outcome was the cumulative incidence of SH initiation in the study units. Secondary outcomes were the proportion of SH renewal at discharge and risk factors for SH initiation and renewal at discharge (patient characteristics, hospital organization). A Cox regression model was used to study risk factors for SH initiation. A mixed effects logistic regression was used to study risk factors for SH renewal at discharge.

**Results:**

SH initiation occurred in 21.5% of participants 20 days after admission. SH renewal at discharge occurred in 38.7% of patients who had initiated it during their stay and were discharged home and in 56.0% of patients discharged to rehabilitation facilities. Neither patients’ characteristics nor hospital organization patterns was associated with SH initiation. SH initiation after the first six days after admission was associated with a lower risk of SH renewal in patients discharged to rehabilitation facilities (OR = 0.19, 95% CI: [0.04–0.80]).

**Conclusions:**

Hospitalization is a period at risk for SH initiation. The implementation of interventions promoting good use of SHs is thus of first importance in hospitals. Specific attention should be paid to patients discharged to rehabilitation facilities.

**Electronic supplementary material:**

The online version of this article (10.1186/s12877-018-0972-3) contains supplementary material, which is available to authorized users.

## Background

According to the International Narcotics Control Board, France ranked 6th for Sedative-Hypnotic (SH) consumption worldwide in 2015 [1]. According to the French National Agency for Medicines Safety, France ranked 3rd in Europe for SH consumption in 2015 with 3.5 million people exposed at least once to hypnotic benzodiazepines (BZDs) or z-drugs (zolpidem, zopiclone) [2]. Regarding the consumption of these drugs in older people, most recent data indicate that 18% of French women and 11% of French men aged 65 and older were prescribed a hypnotic BZD or a z-drug in 2012 [3]. There are no data specifically addressing the consumption of first-generation antihistamines as insomnia medications apart from the consumption of the other SHs. This situation is particularly worrying since hypnotic BZDs, z-drugs and first-generation antihistamines are associated with serious adverse drug events (ADE) in this population, such as falls and cognitive impairment, with an unfavorable benefit-risk balance [4–9]. Importantly, these drugs are cited in several tools as Potentially Inappropriate Medications (Beers, STOPP/START, EU (7)-PIM list) [10–12].

Very few studies addressed SH consumption in the hospital setting. During hospitalization, the prevalence of sleep disorders increases and is reported by 36.7 to 62.7% of hospitalized older patients [13, 14]. Hospitalization could thus be a period at risk for SH initiation. Studies published from 1996 to 2014 report an initiation of SHs for 8.3 to 44.6% of hospitalized patients, all ages combined [15–22]. Moreover, initiation of SHs during hospitalization has been shown to be a risk factor for long-term SH prescription after discharge (OR = 4.65, 95% confidence interval: [1.95–11.09]) [20]. There are no available data specifically about hospitalized older patients in France.

The primary objective of our study was thus to estimate the cumulative incidence of SH initiation among older hospitalized patients in a French hospital setting. Secondary objectives were (1) to estimate the proportion of SH prescription renewal at discharge among patients who initiated it during their stay (2) to study risk factors for SH initiation during hospitalization and (3) to study risk factors for SH prescription renewal at discharge among older patients who initiated it during their hospitalization.

## Methods

### Study design

We conducted a retrospective observational multicenter cohort study.

### Setting

This study retrospectively addressed the period 2016/03/15 to 2016/06/15 in six internal medicine units and six acute geriatric units of eight hospitals located in Paris and its suburbs (France). All types of hospitals were represented: teaching hospitals (*n* = 4), general public hospitals (*n* = 2), non-profit private hospitals (*n* = 1), private clinics (*n* = 1).

### Participants

All patients admitted into participating units during the study period were screened for eligibility. Eligible patients were patients aged 65 and older, with no mention of SH in the “medications taken before hospitalization” section of their hospitalization report and discharged to home or into rehabilitation facilities. Patients transferred to another acute care unit or enrolled in a clinical trial involving SHs were excluded. Patients for whom the prescriptions or the medical chart were unavailable were also excluded.

### Data sources

All data were obtained retrospectively.

Data related to SH prescription during the stay were obtained from the Computerized Physician Order Entry.

Data related to patient characteristics (age, gender), medications taken before hospitalization, reason for admission, length of stay, SH prescription at discharge and type of discharge were obtained from the hospitalization report which is a mandatory document that contains all these data and is inserted in the medical chart at the end of the stay. These data were collected by a trained pharmacist in each center, using a standardized collection grid (Additional file 1).

Data related to hospital organization were collected by a trained pharmacist in each center and obtained from the head nurse of each unit, using a standardized collection grid (Additional file 2).

### Bias related to data sources

This mainly concerns the “medications taken before admission” part of the hospitalization report which is the main data source used for this retrospective study. To guarantee the accuracy of this part of the hospitalization report, we ensured that physicians of the twelve study units used at least three sources of information (patient interview, call to the general practitioner, call to the community pharmacist for example) to complete it. We also conducted an independent pilot study on 50 patients hospitalized in study units: the list of medications taken before admission as written in their hospitalization report was compared to the list of medications taken before admission obtained from these 50 patients’ community pharmacists. The agreement between these two data sources was assessed through the Cohen’s kappa coefficient. With a coefficient of 0.66 the degree of agreement was considered as intermediate, with a tendency for physicians to underestimate medicationss taken before hospitalization in the hospitalization report [23].

### Variables

#### Primary outcome

The primary outcome was SH initiation.

SH definition: prescription of any bedtime medications cited in the following list of SH medications established by the French Health Authority (FHA):

- Long half-life BZD (≥ 20 h as defined by the National French Health insurance system) (Anatomical Therapeutic Chemical classification): Diazepam (N05BA01), Clorazepate (N05BA05), Bromazepam (N05BA08), Clobazam (N05BA09), Prazepam (N05BA11), Nordazepam (N05BA16), Loflazepate (N05BA18), Nitrazepam (N05CD02), Triazolam (N05CD05).
- Short half-life BZD (< 20 h as defined by the National French Health insurance system) (Anatomical Therapeutic Chemical classification): Oxazepam (N05BA04), Lorazepam (N05BA06), Alprazolam (N05BA12), Clotiazepam (N05BA21), Estazolam (N05CD04), Lormetazepam (N05CD06), Loprazolam (N05CD11).
- Z-drugs (ATC classification): Zopiclone (N05CF01), Zolpidem (N05CF02).
- Other (ATC classification): Hydroxyzine (N05BB01), Captodiame (N05BB02), Buspirone (N05BE01), Etifoxine (N05BX03), Alimemazine (R06AD01), Doxylamine (R06AA09), Promethazine (R06AD02).

SH initiation: prescription (systematic or “as needed”) of at least one SH of the above-mentioned list in patients for whom there was no mention of SH in the list of “medications taken before hospitalization” section of their hospitalization report (this section being completed by the physician based on the latest available prescription from primary care before hospitalization).

#### Secondary outcomes

- SH prescription renewal at discharge among patients who had initiated this medication during their stay.
  SH prescription renewal was defined as the mention of prescription of any bedtime SH at discharge on the hospitalization report (either the same as the one prescribed during the stay or another, either systematic or as needed).
- Potential risk factors for SH initiation:
  *Risk factors related to patient characteristics*: age, gender, patients’ type of admission (from home, emergency unit or transfer from another acute care unit), reason for admission as indicated by the physician in the hospitalization report, number of medications taken before hospitalization (total number of active ingredients, including oral, parenteral and topical route, over-the-counter medication, excluding homeopathy and phytotherapy), type of room (single or double) during the stay, medical specialty (internal medicine or geriatric unit).
  *Risk factors related to hospital organization*: type of hospital (general hospital, teaching hospital, non-profit private hospital, private clinic), number of patients per nurse at night, number of patients per nursing assistant at night.
- Potential risk factors for SH prescription renewal at discharge among patients who initiated this medication during their stay:
  *Risk factors related to patients’ characteristics*: age, gender, length of stay, main reason for admission, time between admission and SH initiation, type of discharge (to home, nursing home, rehabilitation facilities).

*Risk factors related to hospital organization*: pharmaceutical review of prescriptions during the stay (pharmaceutical review defined as technical review of the list of patient’s medications (level 1) or review of medications with patient’s full note (level 2) by the pharmacist [24]).

### Statistical analysis

Descriptive analysis: results are presented as mean and standard deviation (sd) or median with interquartile range [IQR] for continuous variables and as absolute number and percentage (%) for categorical variables.

The graphical representation of SH initiation cumulative incidence was performed with the Kaplan Meier approach.

Risk factors for SH initiation: a time to event analysis was performed. Time to event was defined as the duration between hospital admission and the date of SH initiation or date of discharge if no SH initiation. The log-linearity of continuous variables was checked; covariates violating this assumption were transformed into categorical covariates. Proportional hazards assumption was checked plotting the scaled Schoenfeld residuals. Bivariable relationship between each potential risk factor and time to SH initiation was analyzed using a Cox model. Results are presented as Hazard Ratio (HR) and 95% Confidence Interval (CI). Covariates with a *p* value ≤0.20 (log rank test) were entered in the multivariate Cox regression model. A *p* value < 0.05 (likelihood ratio test) in the multivariate model was considered significant.

Risk factors for SH renewal at discharge: In all analyses, the population was stratified based on discharge disposition: home or rehabilitation facilities. Bivariate analyses were performed with the Wilcoxon-Mann Whitney test or the Student test for quantitative variables and the Chi-squared test or the Fisher’s exact test for categorical variables, according to validity conditions of these tests. Variables with a *p* value ≤0.20 were included in the logistic regression model with a random center effect. To avoid convergence failure in the logistic regression model and to respect the assumption of having at least 5 to 10 events per variable, variables having a large number of categories and a very small number of cases within some categories (thus leading to complete or quasi complete separation) were re-scaled with a smaller number of categories. Important variables were forced into the model according to their clinical significance when the p value was ≥0.20. Results are presented as Odds Ratio (OR) and 95% Confidence Interval (CI). *P* values < 0.05 in the logistic regression model were considered statistically significant.

All analyses were performed using R software (version 3.1.2).

## Results

### Study population

**Fig. 1:**
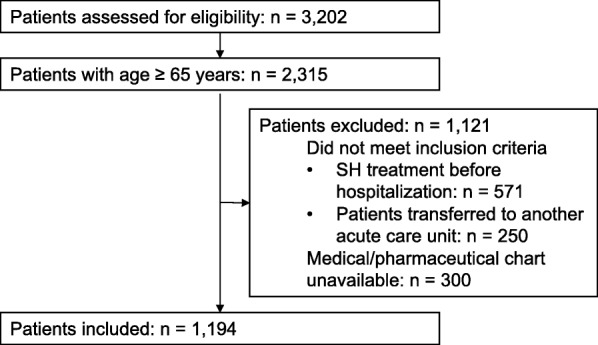
Study flowchart. Legend: SH: Sedative-Hypnotic

Major baseline characteristics of patients are presented in Table 1. Among the 3202 patients admitted to the twelve units during the study period, 2315 were aged 65 and older and 1194 were included in the study. The flowchart with reasons for exclusion is presented in Fig. 1. The mean age of patients was 83.8 ± 8.3 years, 702 were women (58.8%). The mean length of stay was 11.4 ± 7.7 days. The mean number of medications taken before hospitalization was 6.0 ± 3.4. Regarding psychotropic medications taken before hospitalization other than SHs, 19.4% of patients had an antidepressant (*n* = 232), 3.8% had a medication indicated in Alzheimer’s disease (*n* = 45), 2.6% had an antipsychotic (*n* = 31) and 1.9% (*n* = 23) had a major opioid analgesic (morphine, oxycodone, fentanyl).

**Table 1:** Patients’ baseline characteristics (*n* = 1194)

Characteristics	Value
Demographic	
Age, mean ± standard deviation (years)	83.8 ± 8.3
80–89 years old, n (%)	522 (43.7)
≥ 90 years old, n (%)	351 (29.4)
Women, n (%)	702 (58.8)
Type of health facility, n (%)	
Teaching hospital	618 (51.8)
General hospital	262 (21.9)
Non-profit private hospital	228 (19.1)
Private clinic	86 (7.2)
Type of hospitalization unit, n (%)	
Geriatric unit	588 (49.2)
Internal medicine unit	606 (50.8)
Reason for admission, n (%)	
Cardiopulmonary disease	238 (19.9)
Fall	192 (16.1)
Infectious disease	161 (13.5)
Altered General state	139 (11.6)
Neurological disorder	133 (11.1)
Other	331 (27.7)

### Sedative-hypnotic initiation

**Fig. 2:**
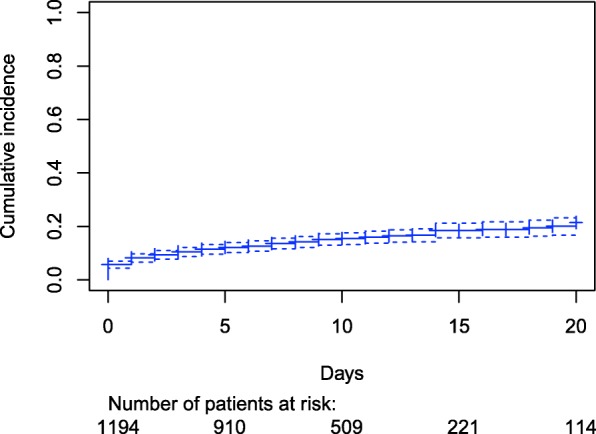
Kaplan Meier graphical representation of SH initiation cumulative incidence. Legend: SH: Sedative-Hypnotic

The median hospitalization duration was 10 days [9-10]. The cumulative incidence of SH initiation is presented in Fig. 2 with a focus on day 0 to day 20 of hospitalization since no initiation occurred after 20 days (maximum duration of hospitalizatio*n* = 77 days). The cumulative incidence of SH initiation two days after admission was 9.4%. It raised to 11.5% 4 days after admission and 21.5% 20 days after admission. The most commonly prescribed SHs were z-drugs (*n* = 116, 62.7%), short half-life BZDs (*n* = 38, 20.5%), hydroxyzine (*n* = 24, 13.0%) and long half-life BZDs (*n* = 7, 3.8%). The SH was prescribed as needed in 42.2% of cases (*n* = 78).

#### Risk factors for SH initiation during hospitalization

Bivariate relationship between each potential risk factor and time to initiation is presented in Additional file 3. Two variables were associated (*p* ≤ 0.20) with the initiation of a SH during the stay: number of medications before admission (HR = 1.04, 95%CI [1.00–1.08], *p* = 0.06] and number of patients per nursing assistant at night (HR_21–40 patients_ = 1.23, 95%CI [0.90–1.68], *p* = 0.20). However, in the multivariable Cox model regression, neither the number of medications before admission (HR = 1.04, 95%CI [1.00–1.09], *p* = 0.05) nor the number of patients per nursing assistant (HR = 1.25, 95%CI [0.91–1.70], *p* = 0.17) remained significantly associated with SH initiation.

### Sedative-hypnotic renewal at discharge

#### Proportion of sedative-hypnotic prescription renewal at discharge

Considering the global population (*n* = 1194), 7.0% of patients aged 65 and older had an initial SH prescription in the hospital and were discharged home with it. Focusing on patients aged 65 and older who initiated a SH during their hospitalization (*n* = 185), the proportion of SH prescription renewal at discharge was 44.9% (*n* = 83). When stratifying the population based on discharge disposition, the proportion of SH renewal at discharge was 38.7% in patients discharged home (*n* = 46/119) and 56.0% in patients discharged to rehabilitation facilities (*n* = 37/66). Among patients discharged home, SH renewal reached 42.2% (*n* = 35/83) in patients aged 80 and older and 38.9% (*n* = 14/36) in patients aged 90 and older. Among patients discharged to rehabilitation facilities, SH renewal reached 53.8% (*n* = 28/52) in patients aged 80 and older and 50.0% (*n* = 12/24) in patients aged 90 and older.

#### Risk factors for SH prescription renewal at discharge

##### Patients discharged home

The length of stay was the only variable associated with the renewal of SH prescription at discharge in the bivariate analysis (*p* = 0.10) (detailed results in Additional file 4). In the logistic regression with random center effect, this variable was not associated anymore with the renewal of SH prescription at discharge. Detailed results are presented in Table 2.

**Table 2:** Risk factors for SH prescription renewal among older patients who had an initiation and were discharged home

Variable	OR [95% CI]	*P* value
Length of stay		
≤ 7 days^a^	1	
8–14 days	1.30 [0.49–3.46]	0.59
> 14 days	2.33 [0.84–6.50]	0.10

*OR*: Odds Ratio (logistic regression with random effect at the center level); *CI*: Confidence Interval; ^a^Reference category; *SH*: Sedative-hypnotics

### Patients discharged to rehabilitation facilities

In this population, the time between admission and SH initiation was the only variable associated with the renewal of SH prescription at discharge in the bivariate analysis (detailed results in Additional file 4). The main result of the logistic regression with random center effect is that when the SH was initiated after the first six days of hospitalization, patients were less likely to be prescribed a SH at discharge (OR = 0.19, 95%CI: [0.04–0.80], *p* = 0.02). Detailed results are presented in Table 3.

**Table 3:** Risk factors for SH prescription renewal among older patients who had an initiation and were discharged to rehabilitation facilities

Variable	OR [95% CI]	*P* value
Time between admission and SH initiation		
≤ 2 days^a^	1	
3–6 days	0.28 [0.05–1.55]	0.14
> 6 days	0.19 [0.04–0.80]	0.02

*OR*: Odds Ratio (logistic regression with random effect at the center level); *CI*: Confidence Interval; ^a^Reference category; *SH*: Sedative-hypnotics

## Discussion

### Key results

This multicenter study assessed SH initiation and prescription renewal at discharge among older adults hospitalized in six geriatric units and six internal medicine units in France. Four days after admission, the cumulative incidence of SH initiation was 11.5%. It raised to 21.5% 20 days after admission. This is in the range of findings from other studies reporting an initiation of SH for 8.3 to 44.6% of hospitalized patients, all ages combined between 1996 and 2014 in Australia, Belgium, Canada, France, Israel and the USA [15–21]. In 83.2% of cases, the SH initiated was a z-drug or a short half-life benzodiazepine, thus in agreement with international guidelines [25, 26]. This is only partly reassuring since these drugs also have a high potential for ADE such as falls [27–29]. The persistence of hydroxyzine and long half-life benzodiazepine prescriptions for 16.8% of patients is also a matter of concern, reflecting a misreading of recommendations and potential ADE related to these drugs.

Another important finding of this study is that the initiation occurred during the first 48 h of hospitalization for 50% of patients, with only 42.2% of prescriptions with the mention “as needed”, suggesting systematic prescriptions at the acute phase of the hospitalization rather than rational prescriptions after non-pharmacological options attempts (e.g. noise and pain control).

This study also addressed the crucial point of SH prescription management at discharge for older patients who initiated this medication during hospitalization. The proportion of SH prescription renewal at discharge achieved 38.7% in patients discharged home and 56.0% in patients discharged to rehabilitation facilities. This is a particularly high proportion compared to two other French studies (population: adult inpatients all ages), one Australian study (population: adult inpatients all ages) and one Canadian study (population: adult aged 65 and over) who reported a proportion of renewal at discharge comprised between 10.1 and 35.7% [16, 18, 19, 22]. According to Zisberg et al., about one third of post-discharge new SH users had the first prescription of it during hospital stay, making hospitalization a turning point for new SH use in older people [20]. This makes our results even more alarming, considering the high cumulative incidence of SH initiation during hospital stay. Moreover, our results show that the final proportion of patients discharged with a new SH prescription comes to 7%, which is considerable compared to the proportion of SH initiation reported by the National French Health Insurance for the community in 2015 that was 1.2% [2].

Beyond these results, identifying specific risk factors for SH initiation and renewal prescription at discharge was of first importance to understand the situation and propose targeted actions to improve it. In our study, the number of medications taken before hospitalization was not significantly associated with an increased risk of SH initiation. However, the HR was 1.04 with a 95% CI comprised between 1.00 and 1.09 (*p* = 0.05). This tends to be in agreement with literature data that report polypharmacy as a risk factor for SH initiation during hospitalization [15, 18]. However, this is of particular concern since these patients are the most at risk for severe ADE. On the contrary, we found no effect of gender, patient type of hospitalization, whereas two studies found an increased risk for women and for patients not coming from home [15, 18].

Surprisingly, none of the following parameters: type of room, number of patients per nurse or per nursing assistant or type of unit (geriatric vs internal medicine) influenced the risk of SH initiation.

Regarding the risk of SH prescription renewal at discharge, a specific attention should be paid to patients discharged to rehabilitation facilities. In this population, the proportion of SH renewal at discharge reached 56.0% whereas it was 38.7% in patients discharged home. This could be explained by the fact that prescriptions may not be as carefully revised as for patients discharged to home. This could also be explained by the fact that unlike patients discharged home that are supposed to recover normal sleep conditions, factors responsible for sleep disorders are expected to persist in patients discharged to rehabilitation facilities [30]. If no specific risk factor could be highlighted for SH renewal at discharge in patients discharged home, an initiation of SH occurring after the first six days of hospitalization was protective (OR = 0.19, 95%CI [0.04–0.80], *p* = 0.02) in patients discharged to rehabilitation facilities. One hypothesis is that, compared to prescriptions occurring during the first two days, the benefit/risk ratio of a SH prescription occurring after the first six days has been well weighed and prescribers are more aware of proper use rules of these medications. Another hypothesis is that the initiation occurred so close to discharge that it was a very punctual prescription and that renewal at discharge was unnecessary. Further prospective studies are needed to underpin these hypotheses. Pharmaceutical review of prescriptions had no influence on renewal at discharge. This may be due to the fact that it only addressed prescriptions during the stay and was not followed up with discharge medication reconciliation by the pharmacist to alert on the risk of SH renewal at discharge.

### Strengths, generalizability and limitations

This study has several strengths: in addition to the large sample size, it addressed different types of hospitals and was implemented in a large geographical area, reflecting an important socio-economic diversity. To our knowledge, this is the first multicenter study to specifically address the cumulative incidence of SH initiation and the proportion of prescription renewal at discharge in hospitalized older patients in France. At the international level, this is also one of the first studies to address risk factors related to hospital organization for SH initiation and renewal at discharge.

However, some limitations must be discussed. First, the retrospective design prevented us from collecting data such as more precise socio-economic characteristics of patients, comorbidities, degree of insomnia, number of SH intakes for patients with “as needed” prescriptions, grade of the prescriber (junior/senior), and precise reason for SH prescription. The lack of comorbidity reporting and precise reason for SH prescription could have led us to overestimate the cumulative incidence of SH initiation since bedtime SHs may have been prescribed to treat anxiety or agitation more than insomnia itself in some cases. However, 62.7% of SHs initiated were z-drugs which are exclusively prescribed in insomnia disorders. Regarding benzodiazepines and antihistamines that can have two indications, the fact that we only considered the one that were prescribed at bedtime exclusively should have limited the risk of classification bias. The fact that 300 potentially eligible patients could not be included due to missing hospitalization report is also a limitation but it is unlikely that the fact that the report was missing was associated with the initiation of SH. The use of the hospitalization report, which is known to slightly underestimate medications taken, to collect history of SH medication before hospitalization could also have led us to overestimate the proportion of patients considered as formerly free from SHs, depending on the quality of charts and patients’ accuracy regarding self-medication reporting. However, as mentioned in the method, the pilot study allowed us to assess this point and showed that the quality of hospitalization reports was sufficient to consider these reports as data sources. Similarly, the fact that 42.2% of SHs initiated were prescribed “as needed” (thus maybe never administered) is potentially another source of overstatement of the actual SH initiation cumulative incidence. Further prospective studies monitoring all these factors are thus needed to confirm our results. Regarding SH renewal at discharge, our objective was to explore risk factors related to patients’ characteristics and hospital organization that were associated with it. Therefore, we did not investigate the impact of these discharge prescriptions on long term consumption of SH: number of refills, adverse events, re-hospitalization for example. Further studies based on the French Health Insurance databases should help us to obtain these data that address the period post discharge and to provide more detailed results.

## Conclusions

This study highlighted that hospitalization is a period at risk for SH initiation in older patients and that the renewal of SHs on discharge prescriptions affected more than half of the patients who initiated this medication during their stay and were discharged to rehabilitation facilities and more than one third of patients discharged home. As recommended by the FHA, the promotion of SH good use should thus be implemented in hospital setting and at the time of discharge. In addition to awareness campaigns directed towards general practitioners in the community, interventions should be implemented in hospitals to reduce both SH initiation and prescription renewal at discharge. If this study did not lead to the detection of specific risk factors regarding SH initiation during hospitalization, it did however highlight that a specific attention should be paid to patients discharged to rehabilitation facilities.

## Additional files

Additional file 1: Data collection grid (patient characteristics). Collection grid for data related to patient characteristics and medications taken before, during and after hospitalization. (PDF 67 kb)

Additional file 2: Data collection grid (hospital organization). Collection grid for data related to hospital organization. (PDF 50 kb)

Additional file 3: Risk factors for sedative-hypnotic initiation among hospitalized patients aged 65 and older: bivariate analysis (Cox regression model). Risk factors for sedative-hypnotic initiation among hospitalized patients aged 65 and older: bivariate analysis (Cox regression model). (PDF 34 kb)

Additional file 4: Risk factors for sedative-hypnotic prescription renewal at discharge among hospitalized patients aged 65 and older who initiated a SH during their stay: bivariate analysis. Risk factors for sedative-hypnotic prescription renewal at discharge among hospitalized patients aged 65 and older who initiated a SH during their stay: bivariate analysis. (PDF 41 kb)

## Abbreviations

ADE: Adverse Drug Event
ATC: Anatomical Therapeutic Chemical classification
BZD: Benzodiazepine
CI: Confidence Interval
FHA: French Health Authority
HR: Hazard Ratio
IQR: Interquartile Range
OR: Odds ratio
Sd: Standard deviation
SH: Sedative-Hypnotic

## References

[1] International Narcotics Control Board: Psychotropic Substances. Statistics for 2015. 2016.https://www.incb.org/documents/Psychotropics/technical-publications/2016/Technical_Publication_2016.pdf. Accessed 24 Nov 2017.

[2] ANSM: État des lieux de la consommation des benzodiazépines en France. 2017.http://ansm.sante.fr/S-informer/Points-d-information-Points-d-information/Etat-des-lieux-de-la-consommation-des-benzodiazepines-Point-d-Information. Accessed 4 Jan 2018.

[3] ANSM: Etat des lieux de la consommation des benzodiazépines en France (online). 2013.http://ansm.sante.fr/var/ansm_site/storage/original/application/3e06749ae5a50cb7ae80fb655dee103a.pdf. Accessed 4 jan 2018.

[4] Glass J, Lanctot KL, Herrmann N, Sproule BA, Busto UE (2005). Sedative hypnotics in older people with insomnia: meta-analysis of risks and benefits. BMJ.

[5] Airagnes G, Pelissolo A, Lavallee M, Flament M, Limosin F (2016). Benzodiazepine misuse in the elderly: risk factors, consequences, and Management. Curr Psychiatry Rep..

[6] Billioti de Gage S, Pariente A, Begaud B (2015). Is there really a link between benzodiazepine use and the risk of dementia?. Expert Opin Drug Saf.

[7] Kaufmann CN, Spira AP, Alexander GC, Rutkow L, Mojtabai R (2017). Emergency department visits involving benzodiazepines and non-benzodiazepine receptor agonists. Am J of Emerg Med.

[8] Shash D, Kurth T, Bertrand M, Dufouil C, Barberger-Gateau P, Berr C (2015). Benzodiazepine, psychotropic medication, and dementia: a population-based cohort study. Alzheimers Dement.

[9] Billioti de Gage S, Moride Y, Ducruet T, Kurth T, Verdoux H, Tournier M (2014). Benzodiazepine use and risk of Alzheimer's disease: case-control study. BMJ.

[10] American Geriatrics Society (2015). American Geriatrics Society 2015 updated beers criteria for potentially inappropriate medication use in older adults. JAGS..

[11] O'Mahony D, O'Sullivan D, Byrne S, O'Connor MN, Ryan C, Gallagher P (2015). STOPP/START criteria for potentially inappropriate prescribing in older people: version 2. Age Ageing.

[12] Renom-Guiteras A, Meyer G, Thurmann PA (2015). The EU (7)-PIM list: a list of potentially inappropriate medications for older people consented by experts from seven European countries. Eur J Clin Pharmacol.

[13] Isaia G, Corsinovi L, Bo M, Santos-Pereira P, Michelis G, Aimonino N (2011). Insomnia among hospitalized elderly patients: prevalence, clinical characteristics and risk factors. Arch Gerontol Geriatr.

[14] Enomoto M, Tsutsui T, Higashino S, Otaga M, Higuchi S, Aritake S (2010). Sleep-related problems and use of hypnotics in inpatients of acute hospital wards. Gen Hosp Psychiatry.

[15] Somers A, Robays H, Audenaert K, Van Maele G, Bogaert M, Petrovic M (2011). The use of hypnosedative drugs in a university hospital: has anything changed in 10 years?. Eur J Clin Pharmacol.

[16] Fagnoni P, Limat S, Haffen E, Henon T, Jacquet M, Sechter D (2007). Does hospitalisation affect hypnotic and anxiolytic drug prescribing?. Pharm World Sci.

[17] Frighetto L, Marra C, Bandali S, Wilbur K, Naumann T, Jewesson P (2004). An assessment of quality of sleep and the use of drugs with sedating properties in hospitalized adult patients. Health Qual Life Outcomes.

[18] Lagnaoui R, Moore N, Longy-Boursier M, Baumevieille M, Begaud B (2001). Benzodiazepine use in patients hospitalized in a department of internal medicine: frequency and clinical correlates. Pharmacoepidemiol Drug Saf.

[19] Howes JB, Ryan J, Fairbrother G, O'Neill K, Howes LG (1996). Benzodiazepine prescribing in a Sydney teaching hospital. Med J Aust.

[20] Zisberg A, Shadmi E, Sinoff G, Gur-Yaish N, Srulovici E, Shochat T (2012). Hospitalization as a turning point for sleep medication use in older adults: prospective cohort study. Drugs Aging.

[21] Gillis CM, Poyant JO, Degrado JR, Ye L, Anger KE, Owens RL (2014). Inpatient pharmacological sleep aid utilization is common at a tertiary medical center. J Hosp Med.

[22] Pek EA, Remfry A, Pendrith C, Fan-Lun C, Bhatia RS, Soong C (2017). High prevalence of inappropriate benzodiazepine and sedative hypnotic prescriptions among hospitalized older adults. J Hosp Med.

[23] Cohen J (1960). A coefficient of agreement for nominal scales. Educ Psychol Meas.

[24] Geurts MM, Talsma J, Brouwers JR, de Gier JJ (2012). Medication review and reconciliation with cooperation between pharmacist and general practitioner and the benefit for the patient: a systematic review. Br J Clin Pharmacoll.

[25] Riemann D, Baglioni C, Bassetti C, Bjorvatn B, Dolenc Groselj L, Ellis JG (2017). European guideline for the diagnosis and treatment of insomnia. J Sleep Res.

[26] Sateia MJ, Buysse DJ, Krystal AD, Neubauer DN, Heald JL (2017). Clinical practice guideline for the pharmacologic treatment of chronic insomnia in adults: an American Academy of sleep medicine clinical practice guideline. J Clin Sleep Med.

[27] Wang PS, Bohn RL, Glynn RJ, Mogun H, Avorn J (2001). Hazardous benzodiazepine regimens in the elderly: effects of half-life, dosage, and duration on risk of hip fracture. Am J Psychiatry.

[28] Wang PS, Bohn RL, Glynn RJ, Mogun H, Avorn J (2001). Zolpidem use and hip fractures in older people. JAGS.

[29] Landi F, Onder G, Cesari M, Barillaro C, Russo A, Bernabei R (2005). Psychotropic medications and risk for falls among community-dwelling frail older people: an observational study. J Gerontol A Biol Sci Med Sci.

[30] Martin JL, Jouldjian S, Mitchell MN, Josephson KR, Alessi CA (2012). A longitudinal study of poor sleep after inpatient post-acute rehabilitation: the role of depression and pre-illness sleep quality. Am J Geriatr Psychiatry.

