# Supplementary material for: Sedative-hypnotic initiation and renewal at discharge in hospitalized older patients: an observational study
Source: BMC Geriatr. 2018 Nov 14;18:278. doi: 10.1186/s12877-018-0972-3 (PMC6234671; doi:10.1186/s12877-018-0972-3)
Supplement: Supplementary file 1 — Data collection grid (patient characteristics). Collection grid for data related to patient characteristics and medications taken before, during and after hospitalization. (PDF 67 kb) [file 12877_2018_972_MOESM1_ESM.pdf]

Additional File 1. Collection grid for data related to patient characteristics and treatment before, during and after hospitalization

HOSPITAL: ……………………………..   **UNIT:** ………………………………….

Inclusion number: …………………..

I. Patient characteristics

Age (date of birth): …………………

Gender: Male ☐   Female ☐

Length of stay in the unit: ………. Days

Hospitalization (dd/mm/yyyy): ………………   **Discharge** (dd/mm/yyyy): ……………….

Type of room: Single ☐   Double ☐

Patient hospitalized from: Home ☐   Emergency unit ☐   Transfer from another acute care unit ☐

Patient discharged to: Home ☐   Nursing home ☐   Rehabilitation structure ☐

Reason for hospital admission: ……………………………………………………………….

II. Medications taken prior to admission

	International Non-proprietary Name		International Non-proprietary Name
**1**		**13**	
**2**		**14**	
**3**		**15**	
**4**		**16**	
**5**		**17**	
**6**		**18**	
**7**		**19**	
**8**		**20**	
**9**		**21**	
**10**		**22**	
**11**		**23**	

III. Sedative-hypnotic initiation during hospitalization (bedtime only)

No ☐

Yes ☐

If yes :

Sedative-hypnotic (SH)treatments prescribed
SH n°1
**International Non-proprietary Name:** ....
**Day of prescription:** …………………………..
**Length of the prescription:** …………………..
**Type of prescription:** as needed ☐ systematic ☐
**Prescription renewal at discharge:** Yes ☐ No ☐
SH n°2 (if applicable)
**International Non-proprietary Name:** ....
**Day of prescription:** ………………………..
**Length of the prescription:** ………………..
**Type of prescription:** as needed ☐ systematic ☐
**Prescription renewal at discharge:** Yes ☐ No ☐
SH n°3 (if applicable)
**International Non-proprietary Name:** ....
**Day of prescription:** …………………………
**Length of the prescription:** …………………
**Type of prescription:** as needed ☐ systematic ☐
**Prescription renewal at discharge:** Yes ☐ No ☐
