# Supplementary material for: Sedative-hypnotic initiation and renewal at discharge in hospitalized older patients: an observational study
Source: BMC Geriatr. 2018 Nov 14;18:278. doi: 10.1186/s12877-018-0972-3 (PMC6234671; doi:10.1186/s12877-018-0972-3)
Supplement: Supplementary file 2 — Data collection grid (hospital organization). Collection grid for data related to hospital organization. (PDF 50 kb) [file 12877_2018_972_MOESM2_ESM.pdf]

Additional file 2. Collection grid for data related to hospital organization.

I. Data related to the hospital:

Name of the hospital : ……………………………………		
Type of hospital:	☐Teaching hospital<br>☐non-profit private hospital	☐General hospital<br>☐private clinic
Number of beds in the hospital: ……………………………		
Type of unit: ☐Internal medicine	☐Geriatric ward	

II. Data related to the medical unit:

Number of beds : ………………………………………………
Occupation rate during the study: …………………………..
Junior prescribers : : ☐Yes ☐No
Number of nurses during the night: …………………………
Number of nursing assistant during the night: ……………………
Number of patients per nurse during the night: …………….
Number of patients per nursing assistant during the night: ………

III. Data related to pharmaceutical review of prescriptions

**Pharmaceutical review of prescriptions**	☐ Yes	☐ No
