# Supplementary material for: Sedative-hypnotic initiation and renewal at discharge in hospitalized older patients: an observational study
Source: BMC Geriatr. 2018 Nov 14;18:278. doi: 10.1186/s12877-018-0972-3 (PMC6234671; doi:10.1186/s12877-018-0972-3)
Supplement: Supplementary file 3 — Risk factors for sedative-hypnotic initiation among hospitalized patients aged 65 and older: bivariate analysis (Cox regression model). Risk factors for sedative-hypnotic initiation among hospitalized patients aged 65 and older: bivariate analysis (Cox regression model). (PDF 34 kb) [file 12877_2018_972_MOESM3_ESM.pdf]

Additional file 3 Risk factors for sedative-hypnotic initiation among hospitalized patients aged 65 and older: bivariate analysis (Cox regression model)

Variables	HR[a]	CI 95 %[a]	P value[b]
**Age**	1.00	0.98 - 1.02	0.95
**Gender**			
Male[†]	1		
Female	0.84	0.62 - 1.13	0.25
**Hospitalized from**			
Home[†]	1		
Nursing home	0.46	0.14 - 1.50	
Transfer from another unit	1.22	0.76 - 1.95	
Emergency unit	0.93	0.65 - 1.33	0.31
**Reason for admission**			
Cardiopulmonary disease[†]	1		
Fall	1.55	0.96 – 2.51	
Infectious disease	1.12	0.64 – 1.93	
Altered general state	1.37	0.80 – 2.32	
Neurological disorder	1.17	0.67 – 2.04	
Other	1.25	0.79 – 1.97	0.57
**Number of medications before admission**	1.04	1.00 – 1.08	0.06[‡]
**Type of health facility**			
General hospital[†]	1		
Teaching hospital	1.34	0.91 – 1.99	
Non-profit private hospital	1.43	0.90 – 2.27	
Private clinic	1.64	0.88 – 3.07	0.31
**Medical specialty**			
Geriatric unit[†]	1		
Internal medicine unit	0.96	0.72 – 1.28	0.77
**Number of patients per nurse at night**			
6-15	1		
16-25	1.12	0.78 – 1.60	
26-35	1.34	0.94 – 1.91	0.25
**Number of patients per nursing assistant at night**			
0-20	1		
21-40	1.23	0.90 – 1.68	0.20[‡]
**Type of room**			
Single room[†]	1		
Double room	0.85	0.63 – 1.14	0.27

HR: Hazard ratio; [†]Reference category; [a]Cox regression model; [b]Log rank test; [‡]Variables selected for the multivariate Cox regression model.
