# Supplementary material for: Sedative-hypnotic initiation and renewal at discharge in hospitalized older patients: an observational study
Source: BMC Geriatr. 2018 Nov 14;18:278. doi: 10.1186/s12877-018-0972-3 (PMC6234671; doi:10.1186/s12877-018-0972-3)
Supplement: Supplementary file 4 — Risk factors for sedative-hypnotic prescription renewal at discharge among hospitalized patients aged 65 and older who initiated a SH during their stay: bivariate analysis. Risk factors for sedative-hypnotic prescription renewal at discharge among hospitalized patients aged 65 and older who initiated a SH during their stay: bivariate analysis. (PDF 41 kb) [file 12877_2018_972_MOESM4_ESM.pdf]

Additional file 4 Risk factors for sedative-hypnotic prescription renewal at discharge among hospitalized patients aged 65 and older who initiated a SH during their stay: bivariate analysis

Variables	Patients discharged home			Patients discharged to rehabilitation facilities		
	SH renewal			SH renewal		
	**No (n = 73)**	**Yes (n = 46)**	**P value**	**No (n = 29)**	**Yes (n = 37)**	**P value**
**Age** (years), median [IQR]	83.8 [12.5]	85.9 [10.3]	0.28[a]	87.0 [7.9]	86.9 [11.7]	0.48[a]
**Gender**, n (%)						
Male	28 (38.4%)	18 (39.1%)	0.93[b]	9 (31.0%)	13 (35.1%)	0.73[b]
Female	45 (61.6%)	28 (60.9%)		20 (69.0%)	24 (64.9%)	
**Length of stay** (days), median [IQR]	10.0 [7.0]	12.0 [8.0]	0.10[a‡]	15.0 [9.0]	12.0 [11]	0.48[a]
**Reason for admission**,n (%)						
Fall	10 (13.7%)	8 (17.4%)	0.91[c]	9 (31.0%)	11 (29.7%)	0.57[c]
Altered general state	10 (13.7%)	7 (15.2%)		4 (13.8%)	4 (10.8%)	
Neurological disorder	6 (8.2%)	3 (6.5%)		7 (24.1%)	5 (13.5%)	
Other	47 (64.4%)	28 (60.9%)		9 (31.0%)	17 (45.9%)	
**Medical specialty**, n (%)						
Geriatric unit	33 (45.2%)	21 (45.7%)	0.96[b]	20 (69.0%)	25 (67.6%)	0.90[b]
Internal medicine unit	40 (54.8%)	25 (54.3%)		9 (31.0%)	12 (32.4%)	
**Time between admission and treatment initiation** (days), median [IQR]	1.0 [4.0]	1.0 [3.0]	0.65[a]	5.0 [9.0]	1.0 [4.0]	0.05[a‡]
**Pharmaceutical analysis of prescriptions**, n (%)						
Yes	56 (76.7%)	34 (73.9%)	0.73[b]	19 (65.5%)	23 (62.2%)	0.78[b]
No	17 (23.3%)	12 (26.1%)		10 (34.5%)	14 (37.8%)	

[a]Wilcoxon-Mann Whitney test; [b]Chi-squared test; [c]Fisher exact test. IQR: interquartile range; SH: Sedative-Hypnotics; [‡]Variables selected for the multivariate analysis.
